# Multicenter European Prevalence Study of Neurocognitive Impairment and Associated Factors in HIV Positive Patients

**DOI:** 10.1007/s10461-017-1683-z

**Published:** 2017-01-31

**Authors:** Lewis J. Haddow, Rosanna Laverick, Marina Daskalopoulou, Jeffrey McDonnell, Fiona C. Lampe, Richard Gilson, Andrew Speakman, Andrea Antinori, Pietro Balestra, Tina Bruun, Jan Gerstoft, Lars Nielsen, Anna Vassilenko, Simon Collins, Alison J. Rodger, For the Cognitive Impairment in People with HIV in the European Region (CIPHER) Study Group

**Affiliations:** 1 Research Department of Infection and Population Health, Mortimer Market Centre, University College London, Capper Street, London, WC1E 6JB UK; 2 Department of Psychology, King’s College London, London, UK; 3 National Institute for Infectious Diseases, Lazzaro Spallanzani, Rome, Italy; 4 Department of Infectious Diseases, Rigshospitalet, Centre for Health & Infectious Disease Research (CHIP), University of Copenhagen, Copenhagen, Denmark; 5 Infektionsmedicinsk Afdeling, Nordsjællands Hospital, Hillerød, Denmark; 6 Belarusian State Medical University, Minsk, Belarus; 7 HIV i-Base, London, UK

**Keywords:** HIV, Prevalence, HIV dementia, Neuropsychological assessment, MCI (mild cognitive impairment), Activities of daily living

## Abstract

We conducted a cross-sectional study in 448 HIV positive patients attending five European outpatient clinics to determine prevalence of and factors associated with neurocognitive impairment (NCI) using computerized and pen-and-paper neuropsychological tests. NCI was defined as a normalized *Z* score ≤−1 in at least 2 out of 5 cognitive domains. Participants’ mean age was 45.8 years; 84% male; 87% white; 56% university educated; median CD4 count 550 cells/mm^3^; 89% on antiretroviral therapy. 156 (35%) participants had NCI, among whom 26 (17%; 5.8% overall) reported a decline in activities of daily living. Prevalence of NCI was lower in those always able to afford basic needs (adjusted prevalence ratio [aPR] 0.71, 95% confidence interval [CI] 0.54–0.94) or with a university education (aPR 0.72, 95% CI 0.54–0.97) and higher in those with severe depressive symptoms (aPR 1.53, 95% CI 1.09–2.14) or a significant comorbid condition (aPR 1.40, 95% CI 1.03–1.90).

## Resumen

Reportamos un estudio de tipo corte transversal que incluye 448 pacientes VIH seropositivos vistos en cinco clínicas especializadas en Europa con el objetivo de medir la prevalencia del trastorno neurocognitivo asociado al VIH (NCI por sus siglas en inglés) y los factores de riesgo asociados a éste. Se usaron pruebas neuropsicológicas computarizadas y en papel para determinar la presencia de NCI, definido como puntuación Z ≤ 1 en al menos 2 de los 5 dominios cognitivos evaluados. La media de edad de los pacientes fue 45,8 años, 84% eran hombres, 87% blancos y 56% tenían educación universitaria. La media de CD4 fue de 550 cel/mm^3^ y 89% de los pacientes recibían terapia antiretroviral. Un total de 156 (35%) participantes tenían NCI, de los cuales 26 (17%, 5,8% de la población de estudio) reportaron deterioro en actividades de la vida diaria. La prevalencia de NCI fue menor en participantes capaces de cubrir sus necesidades básicas (Razón de prevalencia ajustada [aPR] 0,71; Intervalo de confianza del 95% [95% CI] 0,54-0,94) o con educación universitaria (aPR 0,72; 95%CI 0,54-0,97) pero fue mayor en aquellos con síntomas de depresión severa (aPR 1,53; 95%CI 1,09-2,14) o alguna comorbilidad importante (aPR 1,40; 95%CI 1,03-1,90).

## Introduction

HIV-1 can cause neurological disease, but severe impairment is rare in HIV positive (HIV+) patients with access to antiretroviral therapy (ART) [1]. A variety of estimates of prevalence of milder neurocognitive impairment (NCI) have been reported in HIV+ patients, exceeding 50% in studies from Western Europe and North America [2–4], but as low as 19% in other studies using broadly similar neuropsychological (NP) definitions [5, 6]. Estimates may vary because of differences in the characteristics of study populations. Most studies have focused on a particular setting (such as clinical trial participants [5, 7, 8], a single country [2–7, 9], or a specific patient group [6, 10]), and many studies have specifically excluded participants with potentially confounding conditions, detectable viral load or low CD4 count [2, 5, 7, 9, 11]. There is a need to study more diverse HIV + populations inclusive of those with typical comorbid conditions to clarify the effects of clinical, psychological and social variables as risk factors. As well as differences between study populations, differences in assessment method may lead to variations in results. Despite efforts to standardize definitions of HIV-associated NCI, for example with the “Frascati criteria” [12] or with a threshold in global deficit score (GDS) [13], there may be important differences between measuring instruments. The use of computerized neuropsychological testing batteries is one method that may provide stable and cross-cultural measures of cognitive function [14–17].

Our primary aim was to estimate prevalence of NCI, using mainly computerized assessment, in HIV+ outpatient samples in four European countries (UK, Italy, Denmark and Belarus). Our secondary aim was to assess associations of clinical and social variables with NCI, in order to identify potentially modifiable risk factors for NCI in HIV+ patients.

## Methods

### Participants

CIPHER is a five-center European study of HIV+ outpatients (two clinics in London, UK, and one clinic in each of: Copenhagen, Denmark; Minsk, Belarus; Rome, Italy). Participants were sampled opportunistically at the time of routine outpatient attendance from May 2011 to January 2013. Participants had to be at least 18 years of age and able to give consent and complete study procedures. They were recruited regardless of symptoms, treatment and comorbidities.

### Neurocognitive and Functional Status Assessments

The study used Cogstate, software adapted from standard NP tasks for assessment of multiple cognitive domains (http://cogstate.com/academic-2/measurement-of-cognition) [18–20], and two pen-and-paper NP tests. The self-completed tests are mainly non-language-based and the stimuli vary randomly between assessments, reducing the influence of cultural, educational, and practice effects. Computerized NP testing is widespread in research and clinical practice, and several studies have used Cogstate in HIV+ patients in Anglophone countries [5, 14, 15, 21–24], with some work demonstrating good construct validity and correlation with traditional pen-and-paper NP batteries [14, 15, 21]. Ten tests were conducted; these were organized into five domains: psychomotor speed (detection and identification tests); verbal memory (Shopping List learning and recall tests); executive function (Groton Maze learning and recall tests; these tests also assess visuospatial learning and memory); working memory (One-Back speed and accuracy tests); verbal fluency (two pen-and-paper tests: controlled oral word association test [COWAT] and category fluency test [CFT]). The battery was designed to be reliable and standardized across sites, and a single researcher conducted the same training for assessors at all sites. Testing included a full practice run, which was not scored, on the same day as the main assessment. The practice and main assessment took around 90 min in total, not including rest breaks. Analysis of serial testing with Cogstate suggests an early practice effect between the first and second runs, and subsequent stabilization even with short time intervals [25]. The use of a practice run prior to the main assessment optimizes performance while minimizing practice effects and is recommended by the manufacturer’s Research Manual.

Functional status was measured using a self-completed questionnaire, the Modified Lawton & Brody Instrumental Activities of Daily Living Scale, which required participants to indicate their current and best ever level of function on 16 activities of daily living (ADL) such as cooking, shopping, managing finances and taking medication. Where decline had occurred, participants were asked to record the perceived cause as physical, cognitive, or both. The questionnaire has been used in large published studies of HAND [3] and shows good correlation with the Karnofsky score and performance-based measures of function [26].

### Other Data Collection

All participants were attending clinic regularly for HIV care. They completed a questionnaire including demographic, socio-economic and HIV-related factors, depression and anxiety symptom scales (PHQ-9 and GAD-7), medical history, and recreational drug and alcohol use. PHQ-9 scores were graded as: 0–4, none/minimal depression; 5–9, mild; 10–14, moderate; 15–19, moderate-severe; 20–27, severe. GAD-7 scores were graded as: 0–4, none/minimal anxiety; 5–9, mild; 10–14, moderate; 15–21, severe. Problem drinking was defined by the modified (two-question) AUDIT-C questionnaire: a positive score was ≥5 points for men or ≥4 points for women. Financial hardship was assessed by the question “Do you have enough money to cover your basic needs?” Respondents could answer always, most of the time, some of the time, or no, and any answer other than “always” was considered to be positive for this analysis. Additional information collected from clinical and laboratory records at the time of assessment included HIV viral load (VL), CD4 count, ART use, AIDS-defining illnesses, lipids, glucose, liver function tests, and details of neurological, cardiovascular and psychiatric comorbidities. Hepatitis C virus (HCV) status was defined by the most recent HCV RNA assay, or the most recent antibody result in the absence an RNA result.

### Measures and Definitions

Each cognitive test score was converted to an age-, sex-, and education-standardized *Z*-score using normative means and SD. Normative general population data for the computerized tests were provided by Cogstate from Europe, United States of America, South East Asia, Australia and New Zealand, stratified by age group (18–34, 35–50, and 51+ years; minimum cell size n = 145), sex (157 female, 377 male), and education (university/further education, n = 243, or secondary school, n = 291). Exclusion criteria for the normative data included any clinically significant neurological, psychiatric or other disease, impaired visual or auditory acuity, and routine use of neurologically active medications, and assessments had been conducted in the individual’s first language. All or subsets of this normative data had been used previously to classify cognitive function in HIV+ adults [5, 15, 21, 22, 24]. Published age-, sex- and education-stratified norms were used for the COWAT and CFT [27, 28].

Scores for each of the five domains were categorized as severely impaired (*Z* ≤ −2), mildly impaired (−2 < *Z* ≤ −1), or normal (*Z* > −1). NCI was defined as at least mild impairment in at least 2 out of 5 cognitive domains. We excluded participants with fewer than 5 gradable domains. In addition, we defined grades of impairment, based on Frascati criteria: HIV-Associated Dementia (HAD), severely impaired in at least 2 cognitive domains with significant functional decline (decline in ≥4 ADL, reported by the patient to result from cognitive difficulties); Mild Neurocognitive Disorder (MND), NCI with at least mild functional decline (defined as decline in 2–3 ADL) but not meeting criteria for HAD; Asymptomatic Neurocognitive Impairment (ANI), NCI with no functional decline (maximum of one ADL affected). Strict application of the Frascati criteria demands that there is no evidence of a pre-existing cause for the impairment and suggests that assessment be revisited when the comorbid condition has resolved. Therefore, participants with any of a pre-specified list of conditions were noted but not excluded from analysis. The full list of conditions was published as an appendix to the original criteria [12] and includes depressive disorders, historical traumatic brain injury, developmental disabilities, alcohol and substance use disorders, certain HIV-related and non-HIV-related neurologic conditions, and systemic disease likely to affect cognition (including HCV infection). We adopted the nomenclature used by the CHARTER group, where *contributing* conditions were those deemed to have potential for mild cognitive impairment, while *confounding* conditions were those likely to have a substantial effect on cognitive function [3].

### Statistical Analyses

Participant characteristics were compared between countries using the exact test for categorical variables, ANOVA for normally-distributed continuous variables, and the Kruskal–Wallis test for non-normally-distributed continuous variables and ordinal scales. Prevalence of NCI with 95% confidence intervals (CI) was calculated. Modified Poisson regression analyses were used to assess unadjusted and adjusted prevalence ratios for factors associated with NCI, with CIs calculated from robust standard errors [29]. We did not use the Frascati criteria for these analyses because we wished to estimate the associations with factors that would affect the outcome measure (such as depression and confounding conditions). The associations of five variables with NCI were estimated (age, sex, education, and presence of confounding and contributing conditions). Then individual analyses of other clinical and psychosocial variables were performed, with adjustment for age, sex, education and confounding conditions. Finally, all variables found to be associated with NCI (P ≤ 0.1) in either of the earlier models were incorporated into a multivariable analysis. Because depression and anxiety symptom scores were highly collinear, only the stronger of these two variables was included in the final model.

To explore which cognitive domains were driving associations between risk factors and the global measure of NCI, factors associated with the raw scores on each neurocognitive test were assessed in separate models. In these models, the main variables of age, sex, education and confounding conditions and all variables noted to be related to global NCI in subsequent analyses were included in a multivariable model.

## Results

### Study Population

Of 486 patients recruited, 448 (92%) completed both practice and baseline assessments (London, 292 [65%]; Rome 90 [20%]; Copenhagen 42 [9.4%]; Minsk 24 [5.4%]). The remainder were excluded from further analysis: 15 participants who only completed the practice assessment and 23 who did not complete all five cognitive domains. The sample was 87% white, 84% male, and mean 45.8 years of age (SD 9.6). 89% were on ART, of whom 91% had VL < 50 copies/mL (81% overall). The sites differed significantly on many characteristics (Table 1): participants in Minsk had the youngest age, lowest CD4 count, lowest proportion undetectable on ART and shortest time since HIV diagnosis, while Rome had the lowest proportion of university-educated participants, and London had the highest proportion of non-white participants and migrants. Rome and Minsk had a higher proportion of injection drug users, while problem drinking and recent recreational non-injection drug use were particularly prevalent in London. Moderate to severe depressive symptoms were present in a quarter of patients, and anxiety symptoms of similar severity in a fifth. Antidepressants or psychotherapy were being prescribed to 37% of those with moderate/severe symptoms and 10% of those with no or mild symptoms (16% overall).

**Table 1:** Characteristics of HIV positive study participants by site

	London (*n* = 292)	Rome (*n* = 90)	Copenhagen (*n* = 42)	Minsk (*n* = 24)	Total (*n* = 448)	*P value* ^*e*^
Age, mean (SD)	46.3 (9.0)	47.4 (8.2)	46.2 (12.3)	32.3 (6.8)	45.8 (9.6)	<0.001
Male sex, %	92.5	65.6	92.9	41.7	84.4	<0.001
MSM, %	81.2	35.6	83.3	8.3	68.3	<0.001
White ethnicity, %	82.9	91.1	97.6	100	86.8	0.002
Migrant, %^a^	37.0	4.4	4.8	16.7	26.3	<0.001
Can always afford basic needs, %	54.1	48.9	76.2	54.2	55.1	0.025
University educated, %	57.9	35.6	78.6	62.5	55.6	<0.001
Recent psychoactive drug use, %^b^	38.0	14.4	14.3	8.3	29.5	<0.001
Any previous IDU, %	4.5	16.7	4.8	41.7	8.9	<0.001
Problem drinking, %^c^	32.5	5.7	26.2	12.5	25.2	<0.001
Depressive symptoms, %^d^						
Moderate	14.4	6.2	4.8	9.1	11.7	<0.001^f^
Severe	17.2	6.2	7.1	9.1	13.8	
Anxiety symptoms, %^d^						
Moderate	15.2	11.3	4.8	4.4	12.9	0.011^f^
Severe	6.6	7.5	4.8	17.4	7.1	
CD4 count, cells/μL, mean (SD)	597 (252)	555 (265)	659 (239)	367 (184)	582 (256)	<0.001
Nadir CD4 count, median (IQR)	360 (196, 540)	211 (112, 514)	216 (140, 360)	186 (85, 234)	290 (160, 500)	<0.001
Years since HIV+ test, median (IQR)	9.5 (5.3, 15.4)	10.8 (5.5, 19.9)	10.3 (4.1, 19.4)	4.3 (1.6, 8.0)	9.9 (4.8, 16.2)	0.004
On ART, %	87.3	91.1	95.2	95.8	89.1	0.34
HIV RNA <50 copies/mL, %	81.2	77.8	92.9	70.8	81.0	0.09
HCV, %	13.0	18.9	4.8	29.2	14.3	0.024

*ART* anti-retroviral therapy, *HCV* hepatitis C virus, *IDU* intravenous drug use, *IQR* inter-quartile range, *MSM* men who have sex with men

^a^Defined as not born in the country of assessment

^b^Defined as self-reported consumption of opiates for non-medicinal use, amphetamines (including methamphetamine), cocaine, cannabis, ketamine, gamma-hydroxybutyrate, gamma-butyrolactone, psychedelics or mephedrone in the past 3 months

^c^Problem drinking was defined by the modified (two-question) AUDIT-C questionnaire: a positive score was ≥5 points for men or ≥4 points for women

^d^Excludes missing data (n = 12)

^e^P-values are derived from Chi squared or Kruskal–Wallis tests of the null hypothesis that each characteristic or variable is the same across all four sites

^f^Kruskal-Wallis test comparing ordered depression or anxiety symptom scores between sites, using raw scores rather than categories

### Prevalence of NCI and Functional Impairment

Of 448 participants, 156 (35%) met the definition of NCI (at least mild impairment in at least 2 out of 5 cognitive domains). Of those with NCI, 39 (25%) had confounding conditions, the most common being major substance use disorders (n = 22), AIDS-defining conditions of the CNS (n = 8), and severe ongoing depression (n = 12). Of 292 patients not meeting the definition of NCI, 42 (14%) had confounding conditions, including 27 with major substance use, 2 with AIDS-defining CNS conditions, and 9 with severe depression. The spectrum of severity of NCI comprised four (0.9% of the overall study sample; 1.1% of those without confounding conditions) with HAD, 22 (4.9%; 6.0%) with MND, and 91 (20%; 25%) with ANI.

Seventy-five (17%) patients reported a decline in ADL caused by cognitive problems, although just over half of these (42; 56%) did not meet the definition of NCI. Seventy patients (16%) reported a decline in ADL caused by physical problems, and there was significant overlap between self-perceived physical and cognitive impairment, with 42 patients attributing their decline in function to both factors.

### Clinical, Social and Psychological Factors Associated with Prevalence of NCI

Analyses of the main explanatory variables are shown in Table 2. The presence of a confounding condition (prevalence ratio [PR] 1.51, 95% CI 1.15–1.98), being heterosexual and male (PR 2.03, 95% CI 1.53–2.69) or female (PR 1.44, 95% CI 1.04–2.00), and having no university education (PR 1.75, 95% CI 1.35–2.27) were associated with NCI, there was a weak trend associated with increasing age (PR 1.13 per decade, 95% CI 1.00–1.29).

**Table 2:** Unadjusted associations of age, sex, education and comorbid conditions on the prevalence of neurocognitive impairment ^a^

Variable	*n* (%) impaired	Prevalence ratio (95% CI)	*P*
Age, years			
Under 35	18/71 (25.4)	1	
35–50	91/246 (37.0)	1.46 (0.95–2.25)	
51–59	35/104 (33.7)	1.33 (0.82–2.15)	
60+	12/27 (44.4)	1.75 (0.98–3.14)	
Per +10 years		1.13 (1.00–1.29)	0.056
Sexual orientation and gender			
MSM	91/317 (28.7)	1	
Heterosexual men	36/61 (59.0)	2.03 (1.53–2.69)	<0.001
Women (all)	29/70 (41.4)	1.44 (1.04–2.00)	0.029
University educated			
No	91/199 (45.7)	1	
Yes	65/249 (26.1)	0.57 (0.44–0.74)	<0.001
Confounding condition^b^			
No	117/367 (31.9)	1	
Yes	39/81 (48.2)	1.51 (1.15–1.98)	0.003
Contributing condition^b^			
No	131/385 (34.0)	1	
Yes	25/63 (39.7)	1.17 (0.83–1.63)	0.37

*CI* confidence interval

^a^Neurocognitive impairment was defined as s z ≤ −1 in at least 2 out of 5 cognitive domains

^b^Confounding and contributing conditions as defined in published criteria for HIV-associated neurocognitive disorders (12)

Table 3 shows partially and fully-adjusted analyses. University education (adjusted PR [aPR] 0.72, 95% CI 0.54**–**0.97) and always being able to afford basic needs (aPR 0.71, 95% CI 0.54–0.94) were associated with a lower prevalence of NCI. Paradoxically, problem alcohol consumption was also associated with a lower prevalence of NCI (aPR 0.68, 95% CI 0.46–1.00). Being assessed at the Rome study site (aPR 1.85, 95% CI 1.31–2.62), a confounding condition (aPR 1.40, 95% CI 1.03–1.90), and severe depressive symptoms (aPR 1.53, 95% CI 1.09–2.14) were associated with a higher prevalence of NCI. There was weak evidence for higher prevalence of NCI among patients with a longer time since HIV diagnosis (aPR 1.09 per +5 years, 95% CI 0.99–1.19) and non-white ethnic groups (aPR 1.36, 95% CI 0.95–1.97). Analysis of the effects of individual classes of recreational and prescribed drugs found no other correlates of impaired NP function (results not shown). In sensitivity analyses, replacing depressive with anxiety symptoms as a factor in the model did not produce any notable difference in the effect sizes.

**Table 3:** Adjusted associations of psychosocial and clinical variables with prevalence of neurocognitive impairment ^a^

Variable	PR (CI), partially-adjusted^b^	*P*	PR (CI), fully-adjusted^b^	*P*
Age, per +10 years	1.11 (0.97–1.30)	0.12	1.03 (0.87–1.21)	0.75
University education	0.63 (0.49–0.82)	0.001	0.72 (0.54–0.97)	0.028
Confounding condition	1.56 (1.19–2.04)	0.001	1.40 (1.03–1.90)	0.031
Contributing condition	1.12 (0.81–1.56)	0.49	1.00 (0.68–1.47)	0.99
Sexual orientation and gender				
MSM	1		1	
Heterosexual men	1.83 (1.39–2.41)		1.20 (0.83–1.73)	
Women (all)	1.43 (1.02–2.00)	<0.001	0.86 (0.57–1.30)	0.23
Site				
London	1			
Rome	1.60 (1.17–2.19)	0.013	1.85 (1.31–2.62)^c^	<0.001
Copenhagen	1.01 (0.59–1.74)			
Minsk	0.79 (0.37–1.69)			
Non-white ethnicity	1.40 (1.06–1.85)	0.018	1.36 (0.95–1.97)	0.096
Migrant	1.13 (0.88–1.46)	0.34		
Can meet basic needs	0.62 (0.48–0.81)	<0.001	0.71 (0.54–0.94)	0.017
Recent psychoactive drug use	0.82 (0.59–1.13)	0.22		
Lifetime IDU	0.99 (0.70–1.41)	0.96		
Problem drinking^d^	0.56 (0.37–0.83)	0.004	0.68 (0.46–1.00)	0.049
Depressive symptoms^e^				
None/minimal/mild	1		1	
Moderate	1.08 (0.70–1.68)		1.04 (0.66–1.64)	
Severe	1.49 (1.10–2.01)	0.033	1.53 (1.09–2.14)	0.049
Anxiety symptoms^e^				
None/mild	1			
Moderate	1.56 (1.16–2.10)			
Severe	1.21 (0.80–1.84)	0.012		
CD4 count, cells/μL				
>500	1			
351–500	0.89 (0.66–1.21)			
200–350	0.72 (0.47–1.11)			
<200	1.12 (0.71–1.77)	0.40		
Nadir CD4 count				
>500	1			
351–500	0.98 (0.67–1.44)			
200–350	0.85 (0.58–1.24)			
<200	1.05 (0.75–1.48)	0.65		
Years since positive HIV test				
Per +5 years	1.10 (1.01–1.20)	0.037	1.09 (0.99–1.19)	0.090
VL and ART status^e^				
VL <50 copies/mL	1			
Detectable VL on ART	0.96 (0.60–1.54)			
Not on ART	1.10 (0.74–1.64)	0.87		
Hepatitis C positive	0.89 (0.65–1.22)	0.47		
Previous AIDS	0.96 (0.68–1.33)	0.79		

*ART* antiretroviral therapy, *CI* confidence interval, *IDU* intravenous drug use, *MSM* men who have sex with men, *PR* prevalence ratio, *VL* viral load

^a^Neurocognitive impairment was defined as *Z* ≤ −1 in at least 2 out of 5 cognitive domains

^b^The partially-adjusted model included age, sexual orientation, education, and confounding and contributing conditions. The fully-adjusted model included these variables, and all other variables with p ≤ 0.1 in the partially-adjusted model

^c^In the multivariable model, all sites other than Rome were combined into a single reference group

^d^Problem drinking was defined by the modified (two-question) AUDIT-C questionnaire: a positive score was ≥5 points for men or ≥4 points for women

^e^Excludes missing data

Linear regression analyses with the mean *Z* for all tests as a continuous dependent variable broadly identified the same associated factor of ability to meet basic needs (correlation coefficient [β] + 0.31 increase in *Z* score, 95% CI 0.14–0.49), and similar risk factors of duration of HIV infection (β −0.07 per +5 years, 95% CI −0.13 to −0.00), non-white ethnic group (β −0.40, 95% CI −0.68 to −0.11), and study site (β for Rome −0.52, 95% CI −0.75 to −0.29).

### Sensitivity Analyses

When clinic site was removed as a covariate from the fully adjusted Poisson model, lack of university education and being a heterosexual male showed slightly stronger associations with NCI, although there was little overall difference to the pattern of associations. When participants in Italy were excluded, most adjusted estimates changed little, but there was no longer a statistically significant effect of being able to afford basic needs (aPR 0.77, 95% CI 0.52–1.13). To investigate the possibility that some component of the battery was driving the high prevalence of NCI at the Italian site, we compared each test between sites in a linear regression model adjusted for the other important cofactors. Participants in Rome had worse scores on six tests encompassing four out of five cognitive domains (choice reaction time, one-back accuracy, Groton maze executive function task, and verbal learning and recall). On a similar analysis of the Minsk participants, it was noted that the mean *Z* scores for reaction time and choice reaction time tasks were 1.30 and 0.75 lower than the mean for the other three sites, whereas the *Z* scores for verbal learning and recall tasks were >1 SD higher. These slower response times and better verbal learning abilities are difficult to explain and they were only partially attenuated by adjustment for measured confounders. These effects may relate to differences in the technical set-up or the instructions given to participants during the Cogstate battery.

### Factors Associated with Impairment of Specific Neuropsychological Tests

The results of neuropsychological test-specific multivariable analyses are shown in Tables 4 and 5. Only associations with p-values of <0.005 are highlighted, to compensate for multiple hypothesis testing. Some notable patterns emerged. First, increasing age was associated with two timed tasks, with verbal learning and recall, and with the maze executive function task. Second, severe depressive symptoms were associated with slower responses on all timed tasks. Third, most inter-site differences were apparent on language-based tasks (list learning and recall, and verbal fluency), suggesting that although these tests were carried out mainly in the respondent’s language, there are important country-specific differences in the performance of the tests. Fourth, patients who were not always able to meet basic needs were impaired in their performance on the CFT and COWAT. Both of these are heavily vocabulary-dependent and this association may indicate a relationship driven by prior educational attainment or by current communication skills, as well as by executive function (all of which factors could impact on patients’ employment prospects and socioeconomic status).

**Table 4:** Associations between selected factors and scores obtained on single neuropsychological tests where positive numbers suggest cognitive impairment

Variable	Detection speed^a^	Identification speed^a^	One-back speed^a^	Maze learning^b^	Maze recall^b^
Age (per +10 years)	20 (8, 31)	18 (8, 28)^d^	18 (6, 29)^c^	3.8 (1.7, 5.9)^d^	0.7 (0.2, 1.2)
Site					
London	Reference	Reference	Reference	Reference	Reference
Rome	29 (−0, 58)	29 (−5, 53)	19 (−9, 47)	6.1 (1.0, 11.2)	0.5 (−0.8, 1.8)
Copenhagen	10 (−23, 42)	17 (−10, 44)	24 (−7, 55)	−1.6 (−7.3, 4.2)	−0.8 (−2.2, 0.6)
Minsk	106 (56, 156)^d^	54 (12, 96)	38(−10, 86)	−1.1 (−7.8, 9.9)	−0.4 (−2.6, 1.8)
Sexual orientation					
MSM	Reference	Reference	Reference	Reference	Reference
Heterosexual men	−19 (−52, 15)	16 (−11, 44)	−5 (−36, 27)	3.2 (−2.6, 9.1)	1.1 (−0.3, 2.6)
Women (all)	4 (−28, 35)	2 (−24, 28)	3 (−27, 33)	0.25 (−5.3, 5.8)	0.2 (−1.2, 1.6)
University education	−7 (−27, 13)	−2 (−19, 15)	−9 (−28, 11)	−3.2 (−6.7, 0.4)	−1.0 (−1.9, −0.1)
Confounding condition	−4 (−30, 23)	16 (−6, 38)	14 (−11, 39)	2.7 (−2.0, 7.3)	0.0 (−1.1, 1.2)
Contributing condition	2 (−26, 30)	−3 (−26, 21)	0 (−26, 27)	−2.1 (−7.0, 2.9)	−0.9 (−2.1, 0.3)
Non−white ethnic group	40 (9, 72)	24 (−2, 50)	4 (−26, 34)	8.2 (2.7, 13.7)^c^	2.4 (1.0, 3.7)^c^
Cannot always meet basic needs	−2 (−22, 18)	−7 (−23, 10)	−18 (−37, 1)	−2.3 (−5.8, 1.2)	−0.3 (1.2, 0.6)
Depression					
None/mild	Reference	Reference	Reference	Reference	Reference
Moderate	25 (−6, 55)	27 (2, 53)	13 (−16, 42)	−1.5 (−6.9, 3.9)	0.1 (−1.3, 1.4)
Severe	56 (26, 86)^d^	44 (18, 69)^c^	51 (23, 80)^c^	0.4 (−5.0, 5.7)	0.9 (−0.5, 2.2)
Time since HIV positive (per year)	18 (4, 33)	7 (−6, 19)	8 (−6, 22)	1.0 (−1.6, 3.6)	0.8 (0.2, 1.5)
Recent recreational drug use	−4 (−27, 19)	−7 (−26, 12)	−12 (−34, 10)	−1.2 (−5.3, 2.8)	0.1 (−0.9, 1.1)
Problem drinking	10 (−12, 33)	8 (−11, 26)	−0 (−21, 21)	−3.5 (−7.4, −0.5)	−1.2 (−2.2, −0.3)

Tests in this table yield a higher score with worse performance (longer latency or more errors). Associations are expressed as the size of effect on the raw score (95% confidence interval)

^a^Speed tasks are measured in log_10_ (latency in milliseconds) ×10 ^−3^

^b^Maze-based tests of executive function are expressed as total number of errors

^c^P < 0.005

^d^P < 0.001

**Table 5:** Associations between selected factors and scores obtained on single neuropsychological tests where negative numbers suggest cognitive impairment

Variable	One-back accuracy^a^	List learning^b^	List recall^b^	CFT^b^	COWAT^b^
Age (per +10 years)	0.006 (−0.012, 0.024)	−1.2^d^ (−1.6, −0.7)	−0.6^d^ (−0.9, −0.4)	−0.2 (−0.9, 0.5)	1.7 (0. 3, 3.2)
Site					
London	Reference	Reference	Reference	Reference	Reference
Rome	−0.13^d^ (−0.18, −0.09)	−3.5^d^ (−4.7, −2.3)	−1.6^d^ (−2.3, −1.0)	−0.0 (−1.7, 1.7)	1.3 (−2.2, 4.8)
Copenhagen	−0.011 (−0.06, 0.04)	−0.8 (−2.1, 0.5)	−0.8 (−1.4, −0.1)	−0.2 (−2.1, 1.7)	−7.8^d^ (−11.7, −3.8)
Minsk	0.02 (−0.06, 0.09)	1.0 (−1.0, 3.0)	0.6 (−0.5, 1.7)	3.9 (0.9, 6.8)	−2.0 (−8.0, 4.1)
Sexual orientation					
MSM	Reference	Reference	Reference	Reference	Reference
Heterosexual men	−0.06 (−0.11, −0.01)	−2.0^c^ (−3.3, −0.7)	−0.5 (−1.2, 0.2)	−2.1 (−4.1, −0.1)	−5.4 (−9.4, −1.3)
Women (all)	0.00 (−0.05, 0.05)	−0.1 (−1.4, 1.1)	0.4 (−0.3, 1.1)	−0.6 (−2.5, 1.2)	1.1 (−2.7, 4.9)
University education	0.008 (−0.02, 0.04)	0.7 (−0.1, 1.5)	0.3 (−0.1, 0.7)	1.5 (0.3, 2.7)	3.3 (0.9, 5.8)
Confounding condition	0.001 (−0.04, 0.04)	−0.4 (−1.5, 0.6)	−0.5 (−1.0, 0.1)	−2.1 (−3.6, −0.5)	−2.3 (−5.5, 0.9)
Contributing condition	0.01 (−0.03, 0.05)	0.5 (−0.6, 1.6)	0.1 (−0.5, 0.7)	−0.5 (−2.1, 1.2)	2.2 (−1.2, 5.6)
Non−white ethnic group	0.02 (−0.03, 0.07)	−1.6 (−2.8, −0.3)	−0.6 (−1.3, 0.0)	−2.1 (−4.0, −0.3)	−1.3 (−5.1, 2.5)
Cannot always meet basic needs	−0.01 (−0.04, 0.02)	−1.0 (−1.9, −0.3)	−0.4 (−0.9, 0.0)	−2.2 (−3.4, −1.0)^d^	−4.2 (−6.6, −1.8)^d^
Depression					
None/mild	Reference	Reference	Reference	Reference	Reference
Moderate	−0.04 (−0.09, 0.00)	−0.4 (−1.6, 0.8)	−0.2 (−0.9, 0.5)	0.9 (−0.9, 2.7)	−1.8 (−5.5, 2.0)
Severe	−0.05 (−0.10, −0.01)	−0.8(−2.0, 0.4)	−0.7 (−1.4, −0.1)	−0.1 (−1.8, 1.7)	−1.4 (−5.0, 2.3)
Time since HIV positive (per +10 years)	−0.006 (−0.03, 0.02)	−0.1 (−0.7, 0.4)	−0.0 (−0.4, 0.3)	0.4 (-0.5, 1.3)	0.4 (−1.4, 2.2)
Recent recreational drug use	−0.04 (−0.08, −0.01)	−0.8 (−1.7, 0.1)	−0.2 (−0.7, 0.3)	1.0 (−0.4, 2.4)	0.2 (−2.5, 3.0)
Problem drinking	0.04 (0.01, 0.08)	0.4 (−0.5, 1.3)	0.4 (−0.0, 0.9)	0.9 (−0.4, 2.2)	2.7 (-0.0, 5.4)

*CFT* category fluency test, *COWAT* controlled oral word association test

Tests in this table yield a higher score with better performance (better accuracy or more correct responses). Associations are expressed as effect size (95% confidence interval)

^a^One-back accuracy is expressed as the arcsine transformation of the square root of the proportion of correct responses

^b^Verbal fluency tasks and list learning and recall tasks are expressed as total number of valid correct answers

^c^P < 0.005

^d^P < 0.001

## Discussion

In HIV+ patients receiving care at five European HIV clinics, we found that 35% fulfilled standard criteria for at least mild NCI. Of these, a quarter had conditions that confounded a definitive diagnosis of HAND, more than half had no significant decline in daily function, and the remainder (6% of the total sample) had symptomatic HAND. Our estimates of overall prevalence and the proportion with symptomatic impairment are similar to previous estimates [2–4, 7–9], despite important differences in the target population of those studies and the measuring instruments used. The prevalence of self-reported functional decline associated with cognitive difficulties was half that of measured cognitive impairment on the NP battery, and half of the patients perceiving difficulties had normal cognitive function, indicating weak correlation between the two outcomes. The disconnect between objective function and patients’ lived experiences is the subject of a recent study, which concluded that “patient identification of physical versus cognitive causes is poorly associated with objective criteria” [30]. These observations have important implications for classification of HAND in research studies and clinical practice. They also lead to concerns about whether a diagnosis of asymptomatic NCI creates unnecessary anxiety or, as some data suggest [31, 32], heralds future decline.

Limitations to the Frascati criteria, CIPHER study methodology and Cogstate have been discussed in our earlier report on MSM in the UK [23]. In the current analysis, we lacked an HIV-negative control group, and although the normative population data were derived from a healthy sample, we cannot determine whether the high prevalence of NCI is a true effect of HIV infection. Cogstate’s own control data are limited by their origin in an ideal healthy population, but in this respect the platform fares no worse than many traditional neuropsychological tests. The size of the control sample and its distribution according to age, sex and education is adequate for the purpose. Cogstate’s main advantage is its widespread usage which allows comparison with other studies. Another limitation is that our sampling strategy may have been biased and led to over-recruitment of particular social groups, patients with concerns about their neuropsychological health, or those with an interest in this field of research. This study only partially achieves the objective of eliciting risk factors relating to social and cultural differences. Our study sample was less diverse in ethnicity and gender than we anticipated.

An unusual feature of our results was the effect of study site, independent of measured confounders. Our sites differed not only linguistically and culturally but also in their race and gender mix, clinical stage, and prevalence of substance use; this is both a limitation and an interesting finding. The effect of site could have been caused by a real difference in characteristics of participants between HIV+ populations at the different sites, by unmeasured confounders, or by local selection biases or differences in assessment technique. This has implications when comparing data from different locations in other studies. The effects were present despite the fact that Cogstate is designed to be culture-neutral and highly standardized. There were differences between the Rome site and others on several different neurocognitive tests, which is less consistent with this being a problem of administration of some part of the battery, although this cannot be ruled out. In sensitivity analyses, we showed a similar pattern of risk factors while accounting for site effects. Our study included a site from Eastern Europe, which is a relatively under-researched region for the study of neurocognitive disorders and HIV in general. We have endeavored to determine the validity of the Cogstate testing platform in the region, by adjusting for known confounders and also through sensitivity analyses. It appeared that patients in Minsk were slower on reaction time tasks but performed better on verbal memory. It is unfortunate that our final sample from Minsk was small and we hope that future studies can explore these findings in this neglected region.

Several factors were found to be associated with NCI. The effect of time since HIV diagnosis on the prevalence of NCI, while weak in our adjusted analysis, has been reported in the START study in a pre-treatment clinical trial population with CD4 counts above 500 cells/μL [11]. This may reflect chronic neurotoxicity caused by HIV or ART, similar to the effect of nadir CD4 count seen in other patient populations [5, 33]. These observations concur with the Antiretrovirals, Sexual Transmission Risk and Attitudes (ASTRA) study, in which longer time diagnosed with HIV was strongly associated with physical, psychological and functional difficulties [34]. It is not possible, in this study, to separate out the cohort effect of being diagnosed prior to the development of effective ART in the 1990s, and we note that time since HIV diagnosis is only a proxy for time since infection.

Patients with limited economic means, fewer years’ education, and of minority ethnicity were more likely to have NCI, again in keeping with previous work [4, 10, 32, 35–37]. An association between NCI and education was observed despite normalization of *Z*-scores by this factor. This is in keeping with the US Women’s Interagency HIV Study, in which there was a greater adverse effect of low reading level in HIV+ than in HIV-negative women [10]. Residual differences associated with educational attainment and social group may indicate that cognitive reserve is important in HIV-associated NCI [38], or they may relate to inadequate standardization of the normative data. Comorbid conditions have also been identified in other studies as risk factors for NCI in HIV positive patients [8, 35, 36]. Age exerted an effect on several different tasks, particularly speed-based tests, where the primary measure was a latency period in milliseconds, and those involving verbal memory. Severe depression also affected timed tasks and unfortunately there are no standard adjustments available for the assessment of patients with mood disorders.

The paradoxical effect of alcohol in this study, where participants with problem drinking were less likely to have NCI, is at odds with the well-established harmful neurological effects of alcohol. We found a similar effect when the results were re-analyzed with the CAGE questionnaire as a means of measuring problem drinking, and the effect did not disappear after adjustment for potential confounders. Selection bias must be considered as a possible explanation. Information bias in our recording of alcohol use could arise from perceived social desirability, or because the modified AUDIT-C fails to capture cumulative lifetime alcohol consumption. It is notable that alcohol use behavior differed markedly between sites, with the UK and Denmark showing much higher rates of problem drinking than Italy and Belarus.

In conclusion, impaired NP performance was frequently present among HIV + patients, although most patients did not report decline in daily function or they had confounding comorbidities to explain their impairment. The discordance between subjective and objective impairment of cognitive function requires further study. A constellation of factors was associated with poorer test scores, including education, socioeconomic circumstances and time since HIV diagnosis. Important outstanding questions in this field relate to the validity of neuropsychological tests and the Frascati criteria when used across linguistically and culturally diverse groups of HIV+ people. Interventions to prevent NCI in PLWH include earlier diagnosis of HIV, earlier initiation of ART, addressing cardiovascular risk factors, and identifying and treating mental health disorders such as depression and substance use. In future work, the CIPHER study will report how our baseline observations predict longer-term changes in neurocognitive function.
